# Correction: Stämmler et al. Estimating Glomerular Filtration Rate from Serum Myo-Inositol, Valine, Creatinine and Cystatin C. *Diagnostics* 2021, *11*, 2291

**DOI:** 10.3390/diagnostics14171949

**Published:** 2024-09-03

**Authors:** Frank Stämmler, Marcello Grassi, Jeffrey W. Meeusen, John C. Lieske, Surendra Dasari, Laurence Dubourg, Sandrine Lemoine, Jochen Ehrich, Eric Schiffer

**Affiliations:** 1 Department of Research and Development, Numares AG, 93053 Regensburg, Germany; frank.staemmler@numares.com (F.S.); marcello.grassi@numares.com (M.G.); 2 Department of Laboratory Medicine and Pathology, Mayo Clinic, Rochester, MN 55905, USA; meeusen.jeffrey@mayo.edu (J.W.M.); lieske.john@mayo.edu (J.C.L.); 3 Division of Nephrology and Hypertension, Mayo Clinic, Rochester, MN 55905, USA; 4 Division of Endocrinology, Diabetes, Metabolism, and Nutrition, Mayo Clinic, Rochester, MN 55905, USA; dasari.surendra@mayo.edu; 5 Service d’Explorations Fonctionnelles Rénales et Métaboliques, Hôpital Edouard Herriot, 69437 Lyon, France; laurence.dubourg@chu-lyon.fr (L.D.); sandrine.lemoine@chu-lyon.fr (S.L.); 6 Children’s Hospital, Hannover Medical School, 30625 Hannover, Germany; ehrich.jochen@mh-hannover.de

In the original publication [1], there is an error regarding the intercepts in Table 2. The corrected Table 2 appears below. The authors state that the scientific conclusions are unaffected. This correction was approved by the Academic Editor. The original publication has also been updated.

## Figures and Tables

**Table 2:** GFR_NMR_ equation according to serum cystatin C cutoff levels and sex.

Sex	Serum Cystatin C [mg/L]	GFR_NMR_ Equation ^1^
Female	<1.02	238 × cystatin C^−0.4114^ × creatinine^−0.3798^ × valine^0.1628^ × 0.9979^myo_inositol^ × 0.9963^age^
	≥1.02	239 × cystatin C^−0.6443^ × creatinine^−0.3798^ × valine^0.1628^ × 0.9979^myo_inositol^ × 0.9963^age^
Male	<1.22	266 × cystatin C^−0.5867^ × creatinine^−0.3798^ × valine^0.1628^ × 0.9979^myo_inositol^ × 0.9963^age^
	≥1.22	269 × cystatin C^−0.6419^ × creatinine^−0.3798^ × valine^0.1628^ × 0.9979^myo_inositol^ × 0.9963^age^

^1^ Coefficients were rounded to four digits; the sex- and cutoff-dependent intercept was rounded to 0 digits; coefficients are brought to the scale of the respective metabolite serum concentration (µmol for valine, creatinine and myo-inositol; mg/L for cystatin C).
