# Supplementary material for: Estimating Glomerular Filtration Rate from Serum Myo-Inositol, Valine, Creatinine and Cystatin C
Source: Diagnostics (Basel). 2021 Dec 7;11(12):2291. doi: 10.3390/diagnostics11122291 (PMC8700166; doi:10.3390/diagnostics11122291)
Supplement: Supplementary file 1 [file diagnostics-11-02291-s001.zip › diagnostics-1451845-supplementary.pdf]

Supplementary Figures

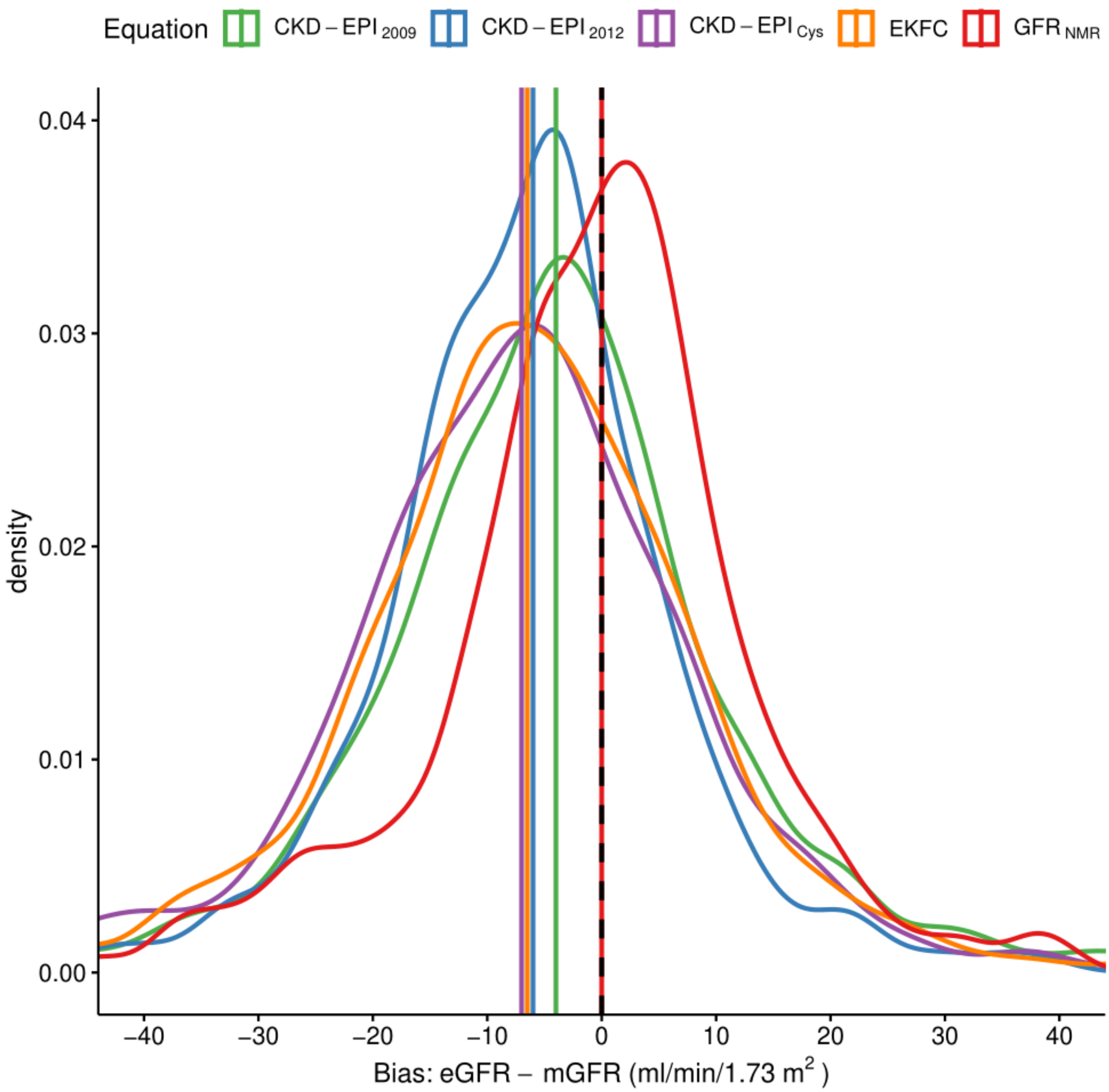

Figure S1. **Distribution of the absolute bias by equation in the external validation set (n=600).** Vertical solid lines indicate the median bias (colored by equation). The black dashed vertical line indicates a bias of zero. As opposed to all other evaluated equations, which tended to underestimate eGFR, GFR_{NMR} showed a median bias of 0 ml/min/1.73 m^2.

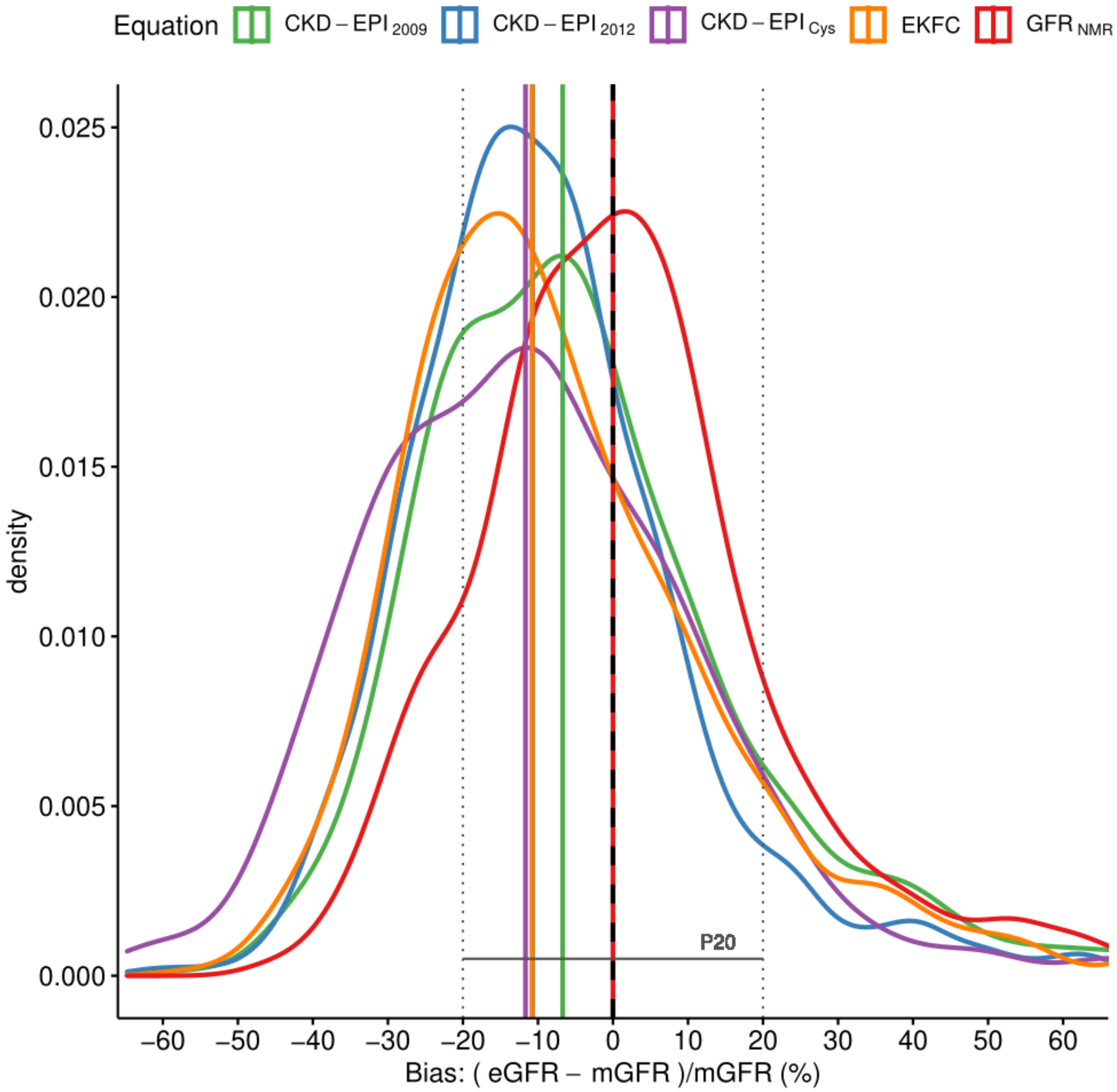

Figure S2. **Distribution of the relative bias by equation in the external validation set (n=600).** Bias (difference between eGFR and mGFR) was expressed relative to mGFR (%). Vertical solid lines indicate the median relative bias (colored by equation). The black dashed vertical line indicates a relative bias of zero. The black dotted lines mark the 20% error tolerance against mGFR (P20). As opposed to other evaluated equations, which underestimated eGFR, GFR_{NMR} showed a median bias of 0%.

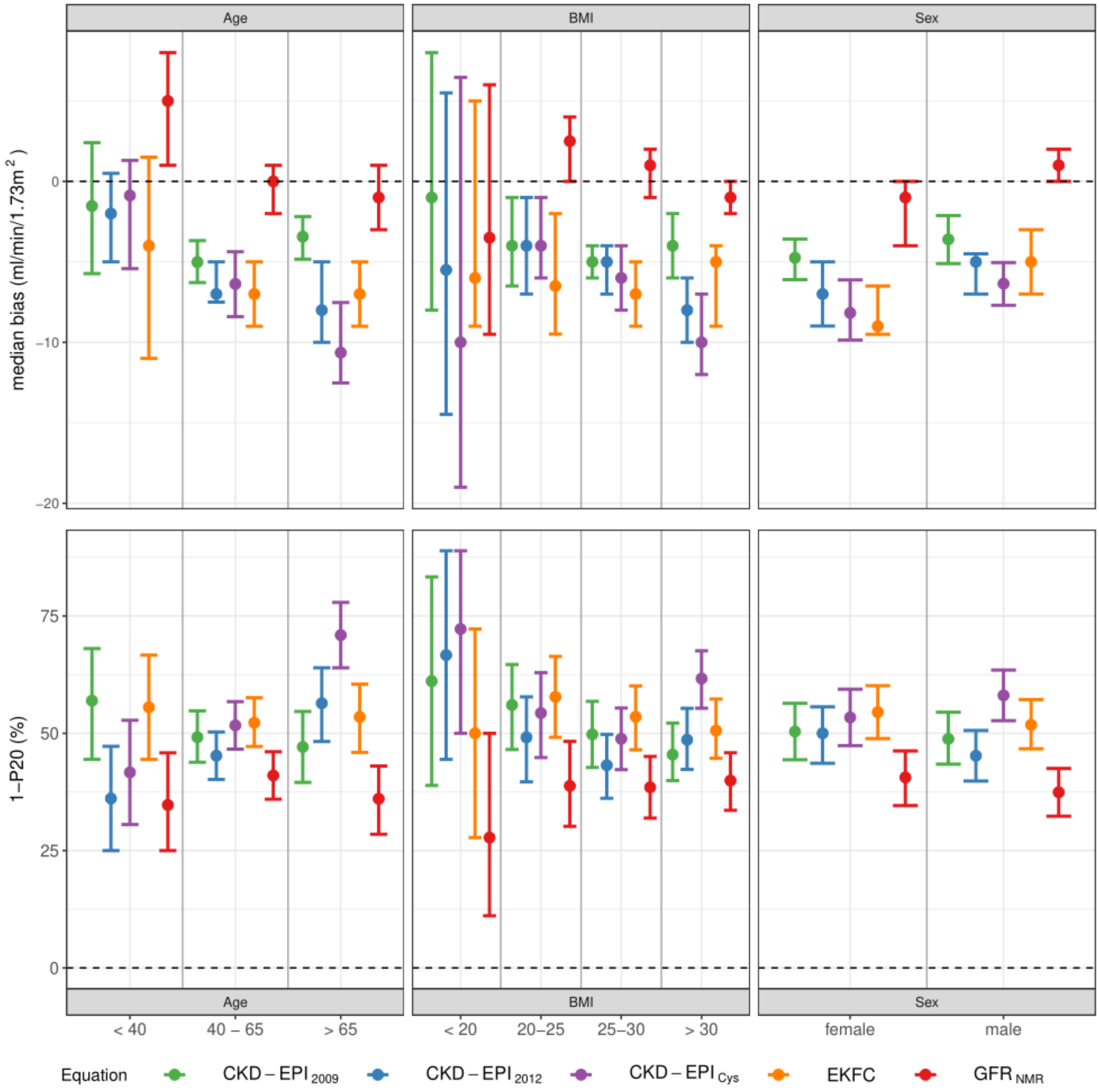

Figure S3. Performance of the eGFR-estimating equations according to age, body mass index (BMI) and sex, in the external validation set (n=600). (a) Median bias ± 95% confidence intervals (CI) according to age groups (<40, 40–65, >65), BMI (<20, 20–25, 25–30, >30) and according to sex (female, male). **(b)** Accuracy (1-P20 ± 95% CI) according to age groups (<40, 40–65, >65), BMI (<20, 20–25, 25–30, >30) and according to sex (female, male). 1-P20 is the percentage of eGFR values lying outside the tolerance range of 20% of measured GFR. The corresponding number of samples and further key performance indicators are described in supplementary tables S1 – S3.

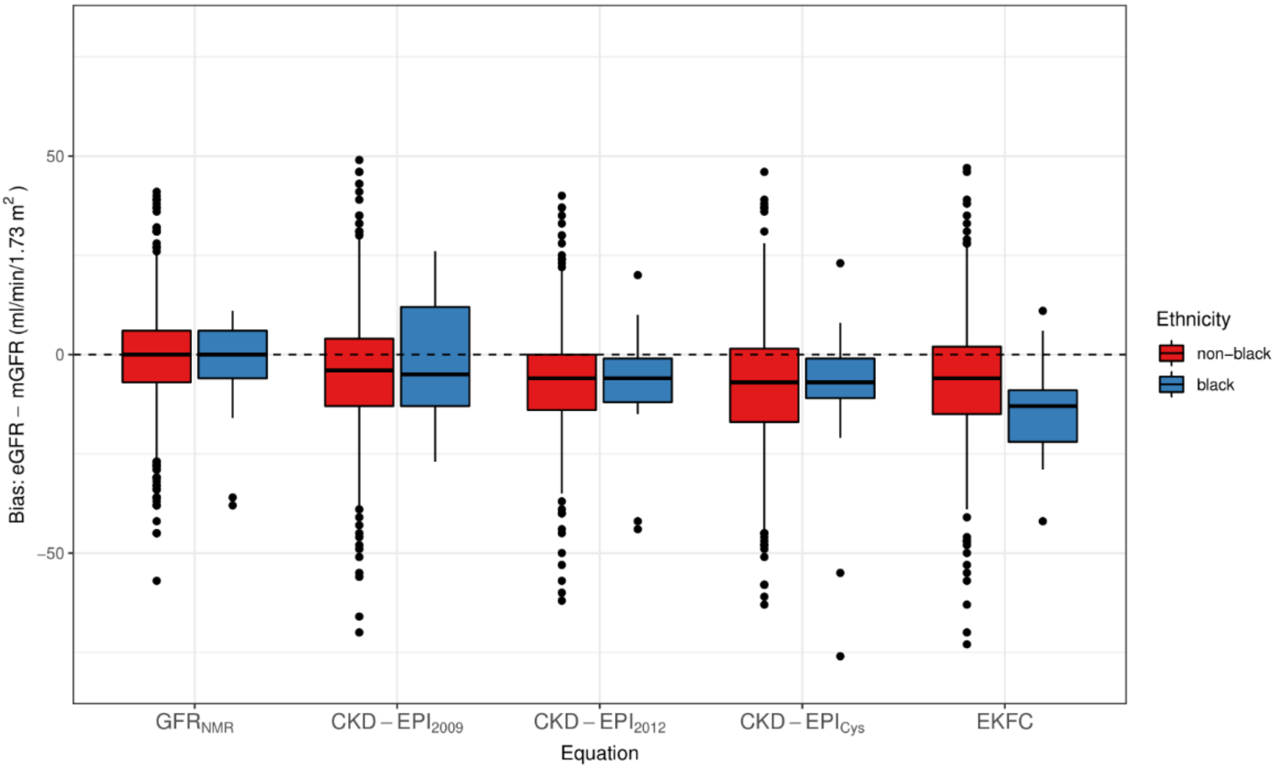

Figure S4. Bias distribution of the eGFR-estimating equations according to ethnicity in the external validation set (n=600). The box plot shows the bias in eGFR estimation by the evaluated equations in black (n = 17) and non-black (n = 583) subpopulations. The black horizontal dashed line indicates a bias of zero. Black horizontal solid lines within the boxes indicate the median bias. Results according to ethnicity should be interpreted with caution due to the under-representation of the black subgroup in the population. Bias appeared comparable in the two subpopulations for most formulas.

Supplementary Tables

Table S1. Performance of eGFR-estimating equations in the external validation set (n=600) according to age.

Variable	Age groups		
	**< 40**<br>n = 72	**40 – 65**<br>n = 356	**> 65**<br>n = 172
**Bias -- median difference (95% CI) [ml/min/1.73 m²]**			
CKD-EPI$_{2009}$	-1.5 (-5.7; 2.4)	-5.0 (-6.3; -3.7)**	-3.4 (-4.8; -2.2)**
CKD-EPI$_{2012}$	-2.0 (-5.0; 0.5)***	-7.0 (-7.5; -5.0)****	-8.0 (-10.0; -5.0)****
CKD-EPI$_{Cys}$	**-0.9** (-5.4; 1.3)**	-6.4 (-8.4; -4.4)****	-10.6 (-12.5; -7.5)****
EKFC	-4.0 (-11.0; 1.5)**	-7.0 (-9.0; -5.0)****	-7.0 (-9.0; -5.0)****
GFR$_{NMR}$	5.0 (1.0; 8.0)	**0.0** (-2.0; 1.0)	**-1.0** (-3.0; 1.0)
**Precision -- IQR of the difference (95% CI) [ml/min/1.73 m²]**			
CKD-EPI$_{2009}$	25.8 (16.5; 34.6)****	16.7 (15.1; 19.6)	13.4 (10.8; 16.2)
CKD-EPI$_{2012}$	16.0 (8.8; 20.5)	**13.2** (11.2; 16.0)	13.0 (11.0; 15.2)
CKD-EPI$_{Cys}$	16.2 (11.8; 22.6)	19.1 (16.6; 21.5)**	13.9 (11.4; 17.6)*
EKFC	24.5 (19.0; 33.0)****	17.0 (15.0; 20.2)	13.0 (10.2; 17.0)
GFR$_{NMR}$	**13.5** (10.0; 18.0)	15.0 (13.0; 17.0)	**11.0** (10.0; 14.0)
**Error -- mean absolute error (95% CI) [ml/min/1.73 m²]**			
CKD-EPI$_{2009}$	15.0 (12.4; 17.6)*	12.4 (11.3; 13.6)**	9.5 (8.3; 10.8)*
CKD-EPI$_{2012}$	**10.1** (8.1; 12.3)	11.6 (10.6; 12.7)**	10.5 (9.4; 11.8)****
CKD-EPI$_{Cys}$	11.8 (9.1; 15.1)	13.4 (12.3; 14.7)****	13.5 (12.1; 15.0)****
EKFC	15.3 (12.5; 18.0)*	13.1 (12.0; 14.3)****	11.1 (9.8; 12.6)****
GFR$_{NMR}$	10.8 (8.8; 12.9)	**10.7** (9.7; 11.8)	**8.3** (7.2; 9.5)
**Accuracy -- 1-P15 (95% CI) [%]**			
CKD-EPI$_{2009}$	56.9 (44.4; 68.1)*	49.2 (43.8; 54.8)*	47.1 (39.5; 54.7)*
CKD-EPI$_{2012}$	36.1 (25.0; 47.2)	45.2 (40.2; 50.3)	56.4 (48.3; 64.0)****
CKD-EPI$_{Cys}$	41.7 (30.6; 52.8)	51.7 (46.6; 56.7)**	70.9 (64.0; 77.9)****
EKFC	55.6 (44.4; 66.7)*	52.2 (47.2; 57.6)**	53.5 (45.9; 60.5)***
GFR$_{NMR}$	**34.7** (25.0; 45.8)	**41.0** (36.0; 46.1)	**36.0** (28.5; 43.0)
**Accuracy -- 1-P20 (95% CI) [%]**			
CKD-EPI$_{2009}$	40.3 (29.2; 51.4)	37.4 (32.6; 42.7)*	35.5 (28.5; 43.0)*
CKD-EPI$_{2012}$	**23.6** (13.9; 33.3)	32.0 (27.2; 37.1)	40.1 (33.1; 47.7)**
CKD-EPI$_{Cys}$	31.9 (22.2; 43.1)	39.3 (34.3; 44.9)**	58.7 (51.2; 66.3)****
EKFC	43.1 (31.9; 55.6)	39.9 (34.6; 45.2)**	40.7 (34.3; 48.2)**
GFR$_{NMR}$	26.4 (16.7; 36.1)	**29.8** (25.0; 34.6)	**26.7** (19.8; 33.7)
**Accuracy -- 1-P30 (95% CI) [%]**			
CKD-EPI$_{2009}$	15.3 (6.9; 23.6)	15.7 (12.1; 19.4)	15.1 (9.9; 20.3)
CKD-EPI$_{2012}$	**9.7** (4.2; 18.1)	**13.2** (9.8; 16.9)	14.5 (9.9; 20.3)
CKD-EPI$_{Cys}$	15.3 (8.3; 23.6)	22.5 (18.3; 27.0)***	26.7 (20.3; 34.3)***
EKFC	13.9 (6.9; 22.2)	16.6 (12.6; 20.5)	16.9 (11.6; 22.7)
GFR$_{NMR}$	13.9 (6.9; 22.2)	13.5 (10.1; 17.1)	**11.0** (6.4; 16.3)

Bold numbers highlight the best performance results in each analysis. 1-P15, 1-P20 and 1-P30 denote the percentage of eGFR values lying outside the tolerance range of 15%, 20% and 30% of measured GFR, respectively. Symbols *, **, *** and **** indicate the level of significance for *p*-values < 0.05, < 0.01, < 0.001 and < 0.0001, respectively, in the pairwise tests against GFR$_{NMR}$ in each subgroup. See also Figure S3. Abbreviations: CI, confidence interval; CKD-EPI, Chronic Kidney Disease Epidemiology Collaboration equations (CKD-EPI$_{2009}$ [1], CKD-EPI$_{2012}$ and CKD-EPI$_{Cys}$ [2]); EKFC, European Kidney Function Consortium equation [3]; GFR, glomerular filtration rate; IQR, interquartile range.

Table S2. Performance of eGFR-estimating equations in the external validation set (n=600) according to sex.

Variable	Sex	
	**female**<br>n = 266	**male**<br>n = 334
**Bias -- median difference (95% CI) [ml/min/1.73 m²]**		
CKD-EPI$_{2009}$	-4.7 (-6.1; -3.6)****	-3.6 (-5.1; -2.1)****
CKD-EPI$_{2012}$	-7.0 (-9.0; -5.0)****	-5.0 (-7.0; -4.5)****
CKD-EPI$_{Cys}$	-8.2 (-9.9; -6.1)****	-6.4 (-7.7; -5.0)****
EKFC	-9.0 (-9.5; -6.5)****	-5.0 (-7.0; -3.0)****
GFR$_{NMR}$	**-1.0** (-4.0; 0.0)	**1.0** (0.0; 2.0)
**Precision -- IQR of the difference (95% CI) [ml/min/1.73 m²]**		
CKD-EPI$_{2009}$	15.1 (13.1; 18.0)	16.8 (15.0; 18.6)
CKD-EPI$_{2012}$	**13.5** (11.8; 16.0)	**14.0** (12.0; 16.0)
CKD-EPI$_{Cys}$	17.5 (14.6; 19.8)	18.9 (16.1; 20.8)**
EKFC	16.0 (13.0; 19.0)	18.0 (16.0; 20.8)**
GFR$_{NMR}$	15.0 (13.0; 17.0)	**14.0** (12.0; 15.0)
**Error -- mean absolute error (95% CI) [ml/min/1.73 m²]**		
CKD-EPI$_{2009}$	12.1 (10.9; 13.5)**	11.7 (10.7; 12.9)***
CKD-EPI$_{2012}$	11.3 (10.2; 12.4)**	11.0 (9.8; 12.0)**
CKD-EPI$_{Cys}$	13.0 (11.7; 14.3)****	13.5 (12.1; 14.7)****
EKFC	13.2 (12.0; 14.6)****	12.4 (11.4; 13.7)****
GFR$_{NMR}$	**10.3** (9.2; 11.5)	**9.8** (8.8; 10.8)
**Accuracy -- 1-P15 (95% CI) [%]**		
CKD-EPI$_{2009}$	50.4 (44.4; 56.4)*	48.8 (43.4; 54.5)**
CKD-EPI$_{2012}$	50.0 (43.6; 55.6)*	45.2 (39.8; 50.6)*
CKD-EPI$_{Cys}$	53.4 (47.4; 59.4)**	58.1 (52.7; 63.5)****
EKFC	54.5 (48.9; 60.2)**	51.8 (46.7; 57.2)***
GFR$_{NMR}$	**40.6** (34.6; 46.2)	**37.4** (32.3; 42.5)
**Accuracy -- 1-P20 (95% CI) [%]**		
CKD-EPI$_{2009}$	37.2 (32.0; 42.9)*	37.1 (32.3; 42.8)**
CKD-EPI$_{2012}$	33.8 (28.2; 39.5)	32.9 (27.8; 38.0)
CKD-EPI$_{Cys}$	42.9 (37.2; 48.5)**	44.9 (39.8; 50.3)****
EKFC	41.7 (35.7; 47.4)**	39.5 (34.4; 44.9)***
GFR$_{NMR}$	**28.9** (23.3; 34.2)	**28.1** (23.4; 32.6)
**Accuracy -- 1-P30 (95% CI) [%]**		
CKD-EPI$_{2009}$	15.4 (11.7; 19.5)	15.6 (11.7; 19.2)
CKD-EPI$_{2012}$	14.7 (10.9; 18.8)	**12.0** (8.7; 15.3)
CKD-EPI$_{Cys}$	24.4 (19.2; 29.3)****	21.6 (17.1; 25.4)**
EKFC	15.8 (12.0; 19.9)	16.8 (12.9; 20.7)
GFR$_{NMR}$	**12.0** (7.9; 15.8)	13.5 (9.9; 17.1)

Bold numbers highlight the best performance results in each analysis. 1-P15, 1-P20 and 1-P30 denote the percentage of eGFR values lying outside the tolerance range of 15%, 20% and 30% of measured GFR, respectively. Symbols *, **, *** and **** indicate the level of significance for p-values < 0.05, 0.01, 0.001 and 0.0001, respectively, in the pairwise tests against GFR$_{NMR}$ in each subgroup. See also Figure S3. Abbreviations: CI, confidence interval; CKD-EPI, Chronic Kidney Disease Epidemiology Collaboration equations (CKD-EPI$_{2009}$ [1], CKD-EPI$_{2012}$ and CKD-EPI$_{Cys}$ [2]); EKFC, European Kidney Function Consortium equation [3]; GFR, glomerular filtration rate; IQR, interquartile range.

Table S3. Performance of eGFR-estimating equations in the external validation set (n=600) according to body mass index (BMI).

Variable	body mass index (BMI)			
	**< 20**<br>n = 18	**20 – 25**<br>n = 116	**25 – 30**<br>n = 213	**> 30**<br>n = 253
**Bias -- median difference (95% CI) [ml/min/1.73 m²]**				
CKD-EPI$_{2009}$	**-1.0** (-8.0; 8.0)	-4.0 (-6.5; -1.0)****	-5.0 (-6.0; -4.0)****	-4.0 (-6.0; -2.0)****
CKD-EPI$_{2012}$	-5.5 (-14.5; 5.5)	-4.0 (-7.0; -1.0)****	-5.0 (-7.0; -4.0)****	-8.0 (-10.0; -6.0)****
CKD-EPI$_{Cys}$	-10.0 (-19.0; 6.5)	-4.0 (-6.0; -1.0)****	-6.0 (-8.0; -4.0)****	-10.0 (-12.0; -7.0)****
EKFC	-6.0 (-9.0; 5.0)	-6.5 (-9.5; -2.0)****	-7.0 (-9.0; -5.0)****	-5.0 (-9.0; -4.0)****
GFR$_{NMR}$	-3.5 (-9.5; 6.0)	**2.5** (0.0; 4.0)	**1.0** (-1.0; 2.0)	**-1.0** (-2.0; 0.0)
**Precision -- IQR of the difference (95% CI) [ml/min/1.73 m²]**				
CKD-EPI$_{2009}$	19.0 (7.5; 47.8)	18.0 (15.2; 25.2)	16.0 (13.0; 18.0)	16.0 (14.0; 18.0)
CKD-EPI$_{2012}$	21.5 (10.0; 36.2)****	17.0 (13.2; 20.0)	**12.0** (9.0; 14.0)	**12.0** (11.0; 16.0)
CKD-EPI$_{Cys}$	26.2 (13.8; 41.0)****	19.2 (14.8; 23.2)*	16.0 (13.0; 19.0)	17.0 (14.0; 20.0)
EKFC	17.5 (3.5; 40.2)	22.5 (16.0; 27.0)****	16.0 (13.0; 20.0)	16.0 (14.0; 19.0)
GFR$_{NMR}$	**16.2** (6.8; 23.5)	**16.0** (12.0; 19.0)	13.0 (11.0; 16.0)	15.0 (12.0; 17.0)
**Error -- mean absolute error (95% CI) [ml/min/1.73 m²]**				
CKD-EPI$_{2009}$	16.8 (9.3; 26.9)	13.3 (11.3; 15.5)**	11.6 (10.2; 13.0)*	11.1 (9.9; 12.4)*
CKD-EPI$_{2012}$	15.0 (9.8; 21.6)	11.0 (9.4; 12.8)	10.4 (9.1; 11.7)	11.5 (10.3; 12.8)****
CKD-EPI$_{Cys}$	16.7 (11.9; 22.2)	12.4 (10.4; 14.8)*	12.4 (11.0; 13.8)***	14.1 (12.8; 15.6)****
EKFC	16.4 (9.8; 26.0)	14.3 (12.2; 16.7)***	12.6 (11.2; 14.0)***	12.0 (10.8; 13.4)***
GFR$_{NMR}$	**11.7** (7.1; 18.6)	**10.0** (8.5; 11.7)	**10.1** (8.9; 11.5)	**9.8** (8.8; 11.0)
**Accuracy -- 1-P15 (95% CI) [%]**				
CKD-EPI$_{2009}$	61.1 (38.9; 83.3)	56.0 (46.6; 64.7)*	49.8 (42.7; 56.8)*	45.5 (39.9; 52.2)
CKD-EPI$_{2012}$	66.7 (44.4; 88.9)	49.1 (39.7; 57.8)	43.2 (36.2; 49.8)	48.6 (42.3; 55.3)*
CKD-EPI$_{Cys}$	72.2 (50.0; 88.9)	54.3 (44.8; 62.9)*	48.8 (42.3; 55.4)*	61.7 (55.3; 67.6)****
EKFC	50.0 (27.8; 72.2)	57.8 (49.1; 66.4)*	53.5 (46.5; 60.1)**	50.6 (44.7; 57.3)*
GFR$_{NMR}$	**27.8** (11.1; 50.0)	**38.8** (30.2; 48.3)	**38.5** (31.9; 45.1)	**39.9** (33.6; 45.8)
**Accuracy -- 1-P20 (95% CI) [%]**				
CKD-EPI$_{2009}$	44.4 (22.2; 66.7)	46.6 (37.1; 56.0)*	34.7 (27.7; 41.3)	34.4 (28.5; 40.7)
CKD-EPI$_{2012}$	61.1 (38.9; 83.3)	34.5 (25.9; 43.1)	29.6 (23.9; 36.2)	34.0 (28.1; 39.9)
CKD-EPI$_{Cys}$	61.1 (38.9; 83.3)	41.4 (31.9; 50.8)	39.0 (32.4; 45.5)*	48.2 (41.9; 54.5)****
EKFC	33.3 (11.1; 55.6)	45.7 (36.2; 55.2)*	40.4 (33.3; 46.9)*	38.7 (33.2; 45.4)**
GFR$_{NMR}$	**16.7** (0.0; 33.3)	**30.2** (22.4; 39.7)	**29.1** (23.0; 35.2)	**28.1** (22.5; 33.2)
**Accuracy -- 1-P30 (95% CI) [%]**				
CKD-EPI$_{2009}$	27.8 (11.1; 50.0)	19.8 (12.9; 27.6)	13.1 (8.5; 17.8)	14.6 (10.3; 19.0)
CKD-EPI$_{2012}$	22.2 (5.6; 44.4)	**13.8** (7.8; 19.8)	**10.3** (6.6; 14.6)	14.6 (10.3; 19.4)
CKD-EPI$_{Cys}$	33.3 (16.7; 55.6)	19.8 (12.9; 27.6)	20.2 (15.0; 25.8)*	25.7 (20.6; 31.6)****
EKFC	22.2 (5.6; 44.3)	26.7 (19.0; 35.3)	13.1 (8.9; 17.8)	13.8 (9.5; 17.8)
GFR$_{NMR}$	**16.7** (0.0; 33.3)	17.2 (10.3; 25.0)	10.8 (7.0; 15.0)	**12.3** (8.3; 16.6)

Bold numbers highlight the best performance results in each analysis. 1-P15, 1-P20 and 1-P30 denote the percentage of eGFR values lying outside the tolerance range of 15%, 20% and 30% of measured GFR, respectively. Symbols *, **, *** and **** indicate the level of significance for *p*-values < 0.05, < 0.01, < 0.001 and < 0.0001, respectively, in the pairwise tests against GFR$_{NMR}$ in each subgroup. See also Figure S3. Abbreviations: CI, confidence interval; CKD-EPI, Chronic Kidney Disease Epidemiology Collaboration equations (CKD-EPI$_{2009}$ [1], CKD-EPI$_{2012}$ and CKD-EPI$_{Cys}$ [2]); EKFC, European Kidney Function Consortium equation [3]; GFR, glomerular filtration rate; IQR, interquartile range.

References

1. Levey, A.S.; Stevens, L.A.; Schmid, C.H.; Zhang, Y.L.; Castro, A.F.; Feldman, H.I.; Kusek, J.W.; Eggers, P.; Van Lente, F.; Greene, T.; et al. A New Equation to Estimate Glomerular Filtration Rate. *Ann Intern Med* **2009**, *150*, 604–612, doi:10.7326/0003-4819-150-9-200905050-00006.
2. Inker, L.A.; Schmid, C.H.; Tighiouart, H.; Eckfeldt, J.H.; Feldman, H.I.; Greene, T.; Kusek, J.W.; Manzi, J.; Van Lente, F.; Zhang, Y.L.; et al. Estimating Glomerular Filtration Rate from Serum Creatinine and Cystatin C. *N Engl J Med* **2012**, *367*, 20–29, doi:10.1056/NEJMoa1114248.
3. Pottel, H.; Björk, J.; Courbebaisse, M.; Couzi, L.; Ebert, N.; Eriksen, B.O.; Dalton, R.N.; Dubourg, L.; Gaillard, F.; Garrouste, C.; et al. Development and Validation of a Modified Full Age Spectrum Creatinine-Based Equation to Estimate Glomerular Filtration Rate : A Cross-Sectional Analysis of Pooled Data. *Ann Intern Med* **2021**, *174*, 183–191, doi:10.7326/M20-4366.
